# Methodology for integrated analysis of vector- and spectroscopic bioimpedance methods

**DOI:** 10.2478/joeb-2024-0018

**Published:** 2024-12-17

**Authors:** José Luis García Bello, Alcibiades Lara Lafargue, Héctor Camué Ciria, Taira Batista Luna, Yohandys Zulueta Leyva

**Affiliations:** 1 Autonomous University of Santo Domingo (UASD) Dominican Republic, Santo Domingo, Dominican Republic; 2 National Center for Applied Electromagnetism (CNEA). Universidad de Oriente. Santiago de Cuba, Cuba; 3 Departamento de Física, Facultad de Ciencias Naturales y Exactas, Universidad de Oriente. Santiago de Cuba, Cuba

**Keywords:** Bioimpedance, tolerance ellipses, body composition

## Abstract

Electrical bioimpedance is based on the opposition exerted by body tissues to the passage of an electrical current. This characteristic allows the assessment of the individual's body composition, nutritional status, and hydration status. Electrical bioimpedance can be used to estimate body composition, health-related markers, general health status, diagnosis and prognostic of diseases, evaluation of treatment progress, and others. The aim of this study is to propose a methodology that allows us to integrate two methods of electrical bioimpedance analysis: bioelectrical impedance vector analysis, and bioelectrical impedance spectroscopy to evaluate the health of individuals.

For methodology validation a retrospective clinical investigation was carried out where the data of healthy individuals and cancer patients included in the Database of the characterization of bioelectrical parameters by electrical Bioimpedance methods were analyzed.

The values of electrical resistance and electrical reactance are higher in cancer patients compared to healthy individuals. However, the phase angle is lower in these patients. In the advanced stages of the disease, patients are located outside the tolerance ellipses. All these results are obtained at the characteristic frequency.

The integration of bioelectrical impedance vector analysis, and bioelectrical impedance spectroscopy can be a sensitive complementary tool, capable of establishing differences between healthy individuals and cancer patients. Enrichment could be achieved by including the analysis of different physiological parameters through estimation equations validated by BIS parameters.

## Introduction

Bioelectrical impedance (BIA) is an indirect, non-invasive, and inexpensive method that consists of applying an electrical stimulus (alternating electric current) through emitting electrodes and observing the response through receiving electrodes in a defined frequency range. This allows direct measurement of the electrical properties of tissues in real time [1–3]. These aspects are considered areas of interest to monitor the health and well-being of the individual [4,5].

In general, BIA can be used to estimate body composition, to track health-related markers, including hydration and malnutrition, general health status, diagnosis and prognostic of diseases, evaluation of treatment progress, athletes and others [6].

There are several modalities for the application of bioimpedance. Depending on the frequency applied, this technique is classified into single frequency BIA (SF-BIA) [7], multifrequency BIA (MF-BIA) [8], bioelectrical impedance spectroscopy (BIS) [9], and we also have the bioelectrical impedance vector analysis (BIVA) [10]. The SF-BIA modality uses a single frequency of 50 kHz to estimate total body water (TBW) and fat-free mass (FFM). Still, it cannot determine intracellular water (ICW) [11,12].

The MF-BIA variant attempts to estimate ICW and extracellular water (ECW) by measuring various frequencies through different mathematical models. However, MF-BIA models also have significant limitations since body mass (BM) is required as an independent variable [13,14]. BIS is another bioimpedance method whose primary purpose is hydration measurement. This method can also be used for other body composition values [15,16].

Due to the limitations of these methods, alternative techniques such as BIVA emerged, which do not estimate body tissues or fluids [17]. In this modality, the optimum frequency of 50 kHz is used to calculate the electrical resistance (R), the capacitive reactance (Xc), and the phase angle (Φ) [18,19].

Bioimpedance is an attractive tool for studying the electrical behavior of an organism whose electrical properties are closely related to its composition and structure [20]. This fact makes it possible to obtain information on the organism's physiological state [21]. In this sense, it is relevant to determine characteristic values of bioimpedance as a function of frequency or impedance spectrum. This technique allows us to determine various tissue conditions due to pathological and physiological conditions [22].

The objective of this work is to propose a new methodology that integrates the best characteristics of applications in clinical practice of two methods of electrical bioimpedance analysis, BIVA and BIS, into the characteristic frequency, in healthy individuals, and cancer patients, of both sexes.

## Materials and methods

### Integrated analysis at characteristic frequency

For the characterization and graphic analysis of the bioelectrical and anthropometric parameters in healthy individuals and cancer patients, a methodology that allows the integration of the two methods: BIVA and BIS was designed. Graphs of the bioelectrical parameters electrical resistance (R) and capacitive reactance (Xc) are normalized according to the subject's height (H), obtaining R/H and Xc/H, both in Ω/m. The analysis was carried out in the characteristic frequency of the individuals.

As a first step, using BIS, n measurements are performed in a frequency range and using the real part of the following mathematical model:

1 $$Z(\omega )=R_{\infty }+\frac{R_{0}-R_{\infty }}{1+{\left(j\omega \tau \right)}^{\alpha }}$$

where Z (w) is the complex impedance, R_∞_ is the electrical resistance when the frequency tends to infinity, R_0_ represents the electrical resistance when the frequency tends to zero, $j=\sqrt{-1}$ , ω represents angular frequency, τ is the average time constant, and α is an empirical parameter characteristic of the distribution of the relaxation frequency [23,24].

By adjusting the measurements of the resistive components resulting from the observations made, we obtain:

2 $$R(\omega )=R_{\infty }+\frac{R_{0}-R_{\infty }}{1+{\left(\omega^{2}\tau^{2}\right)}^{\alpha }}$$

where R(ω) is the electrical resistance as a function of frequency.

Next, the parameters τ and ω_c_ are estimated. ω_c_ is given by:

3 $$\omega_{c}=\frac{1}{\tau }$$

where ω_c_ is the characteristic angular frequency, represented by:

4 $$\omega_{c}=2\pi f_{c}$$

where *f*_c_ is the characteristic frequency in Hz.

The imaginary component is expressed by equation (5):

5 $$X_{c}=-\frac{\omega \tau \left(R_{0}-R_{\infty }\right)}{1+{\left(\omega^{2}\tau^{2}\right)}^{\alpha }}$$

In addition, Rc (characteristic electrical resistance) and Xcc (characteristic electrical reactance), R_0_ and R_∞_ at ω_c_, are estimated with BIS.

In the second step the Rc and Xcc parameters are normalized with the subject's height, obtaining Rc/H and Xcc/H. These parameters together with standard deviation, correlation between the variables, the number of observations, and sex are used as input variables for BIVA program [25].

After this, the patterns of the 50th, 75th, and 95th percentile tolerance ellipses are established for a reference population in the characteristic frequency, according to age group and sex.

In the third step, the R/H and Xc/H measurements at the characteristic frequency overlap within the tolerance ellipse.

In the fourth step, for the graphical analysis each quadrant is divided into smaller zones, according to the position of the individual vector concerning the 50th, 75th, and 95th percentiles. In this case, I-1 indicates that the vector is in the first quadrant (thin individuals), within 50 and 75% (healthy individuals); I-2 and I-3 reveal that it is in the same quadrant, but at 95% or outside of it, respectively. This is valid for the rest of the quadrants. Individuals whose vectors are found in I-1, II-1, III-1, IV-1, are considered healthy; while those found in I-2, II-2, III-2, IV-2 probably present some pathology. Those located in I-3, II-3, III-3, and IV-3 may have associated pathologies.

### Validation of the methodology

#### Subject recruitment

An analytic, retrospective, and cross-sectional investigation was carried out using the Database of the characterization of bioelectrical parameters by methods of electrical Bioimpedance [26], which included healthy individuals (55 female and 81 male) and cancer patients (24 female and 18 male).

#### Bioimpedance measurements

Using a BioScan_®_ 98 model Bioimpedance analyzer from Biologica Tecnología Médica S.L., Barcelona, Spain, we gathered bioimpedance parameters through the tetrapolar whole-body configuration. The volunteers, who had fasted for a minimum of 3 h, emptied their bladders, and abstained from exercise and alcohol for the preceding 12 h, were part of the study. Measurements were taken at a frequency range of 10 to 250 kHz (in our study the number of points measured was 60, n=60) using Ag/AgCl electrodes model 3 M Red Dot 2560.

The study took place in a room with air conditioning set to 23°C and a relative humidity between 60–65 %.

Volunteers were asked to lie down on a non-conductive surface, without any clothing or a pillow under their heads. Their arms were positioned 30° away from their chest and their legs were spread apart at 45°. The injector electrodes were positioned (after cleaning the skin with 70 % alcohol) on the inner side of the dorsal surfaces of the hands and feet, near the metatarsophalangeal and third metacarpal joints. The detector electrodes were placed between the distal ends of the ulna and radius, at the level of the pisiform prominence, and also at the midpoint between both malleoli. During the measurements, a 5 cm gap between detector and injector electrodes was used.

Body weight and height were measured with minimal clothing and barefoot using a Health Scale (SMIC, China). The margin of error for weight and height measurements was 0,1 kg and 0,5 cm, respectively. Each subject stood with their feet together, heels and spine resting against the instrument to ensure accurate readings.

#### Information processing

For statistical analysis, the SPSS version 25 program (SPSS Inc., Chicago, Illinois, USA) for Windows was used.

#### Ethical approval

The data collection considered all relevant national regulations and institutional policies and the tenets of the Helsinki Declaration and has been revised and approved by the ethical committee and scientific council of Centro Nacional de Electromagnetismo Aplicado, Universidad de Oriente, Cuba.

## Results

**Fig. 1:**
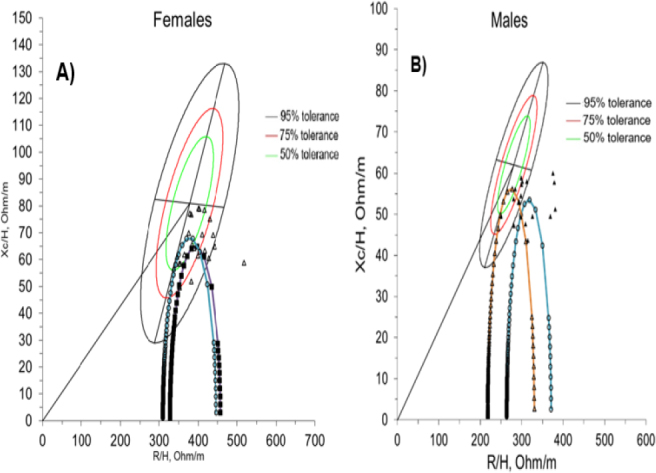
Ellipses of tolerances of a healthy population at the characteristic frequency. A) female individuals (n = 24): (Δ) patients with cancer located in the fourth quadrant (Q IV); (■) average Cole's arc of the group of patients belonging to the fourth quadrant with a maximum value within the 75% ellipse (located Q IV-2); (○) Average reference Cole's arc of a healthy female population belonging to the fourth quadrant, whose maximum value is located within the 50% ellipse (Q IV-1). B) Male individuals (n = 18): (▲) patients with cancer located in the fourth quadrant (Q IV); (○) average Cole's arc of the patient group belonging to the fourth quadrant with a maximum value outside the 95% ellipse (Q IV-3); (Δ) Average reference Cole's arc of a healthy male population belonging to the fourth quadrant, whose maximum value is located within the 50% ellipse (Q IV-1).

Figure 1 shows the behavior of male and female cancer patients. Figure 1a shows that out of the 24 female patients, 22 are located fundamentally within the tolerance ellipses of 50th, 75th, and 95th percentiles; and two are located outside the area corresponding to these ellipses.

However, Figure 1b shows that of the 18 male patients, 9 are located within the tolerance ellipses of the 50th, 75th, and 95th percentile, and the remaining 9 are located outside the area corresponding to these ellipses. Patients of both sexes are observed in the fourth quadrant of the corresponding tolerance ellipses 50th and 75th percentile (regions belonging to healthy individuals).

Tables 1 and 2 present the values of the bioelectrical and anthropometric parameters of healthy individuals and male and female cancer patients, respectively, obtained by BIS. In both cases, cancer patients belong to the fourth quadrant of the tolerance ellipses (for the 50th, 75th, and 95th percentile).

**Table 1.** Bioelectrical and anthropometric parameters of healthy individuals and female cancer patients in the fourth quadrant of the tolerance ellipses (for the 50th, 75th, and 95th percentile) obtained by BIS.

Parameters	Cancer Patients N = 24		Healthy subjects N = 55	
	Mean	Sd	Mean	Sd
Age (years)	53,04	14,56	34,00	12,38
Weight (kg)	60,22	11,80	57,62	8,72
Height (m)	1,57	0,06	1,57	0,07
Zc (Ω)	627,55	52,04	602,22	41,06
R_0_ (Ω)	721,33	55,27	699,56	47,58
R_∞_ (Ω)	516,65	52,61	485,57	36,05
Fc (kHz)	42,80	7,09	44,83	5,38
Φc	9,43	1,27	10,24	0,92

1 N: number of individuals; Sd: standard deviation; Zc: characteristic electrical impedance; R_0_: electrical resistance at low frequency; R_∞_: electrical resistance at high frequency; Fc: characteristic frequency; Φc: characteristic phase angle.

**Table 2.** Bioelectrical and anthropometric parameters of healthy individuals and male cancer patients located in the fourth quadrant of the tolerance ellipses (for the 50th, 75th, and 95th percentile) obtained by BIS.

Parameters	Cancer Patients N = 18		Healthy subjects N = 81	
	Mean	Sd	Mean	Sd
Age (years)	50,89	14,59	21,51	8,67
Weight (kg)	71,07	25,59	65,82	6,62
Height (m)	1,66	0,09	1,70	0,05
Zc (Ω)	535,03	70,85	475,89	39,86
R_0_ (Ω)	616,46	74,60	561,80	45,43
R_∞_ (Ω)	438,43	69,00	370,40	35,20
Fc (kHz)	40,80	7,66	42,53	2,33
Φc	9,69	1,26	11,63	0,94

1 N: number of individuals; Sd: standard deviation; Zc: characteristic electrical impedance; R_0_: electrical resistance at low frequency; R_∞_: electrical resistance at high frequency; Fc: characteristic frequency; Φc: characteristic phase angle.

The results of both tables show that, for both sexes, the values of electrical resistance at low frequency (R_0_), at high frequency (R_∞_), and characteristic electrical impedance (Zc) were higher in patients with suspected cancer compared to healthy individuals of the same sex. However, the characteristic phase angle values (Φc) were lower in patients with suspected cancer.

### Characterization of subjects with a diagnosis of the disease confirmed by anatomical pathology laboratory

**Fig. 2:**
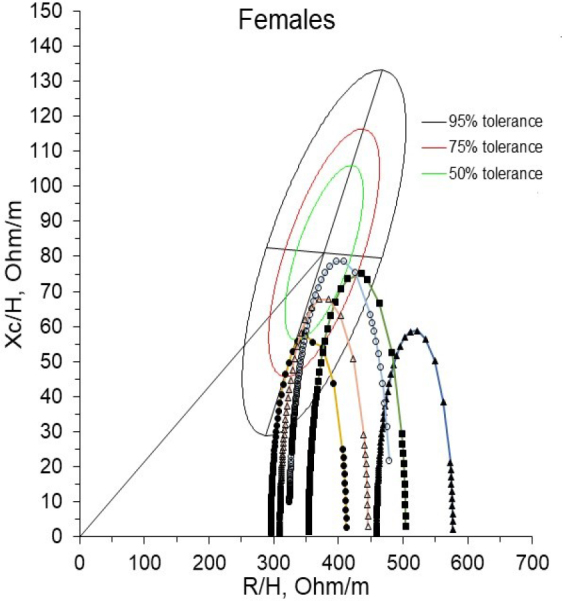
Ellipses of tolerances of a healthy female population at the characteristic frequency; (Δ) reference Cole's arc of a healthy female population, belonging to the fourth quadrant with a maximum value located within the 50% ellipse (Q IV-1). Cole's arches in four female patients diagnosed with cancer, by anatomical pathology laboratory: (•) Cole's arc of a patient with cervical neoplasia (stage Ib), with a maximum value located in the fourth quadrant and within the 75% tolerance ellipse (Q IV-1); (○) Cole's arc of a patient with breast neoplasm (stage IIIb), with maximum value located in the fourth quadrant quadrant and within the 50% tolerance ellipse (Q IV-1); (▲) Cole's arc of a patient with cervical neoplasia (stage IIIb), with a maximum value located outside 95% tolerance ellipse (Q IV-3); (■) Cole's arc of a patient with endometrial cancer (stage IIb), with a maximum value located in the fourth quadrant and within the 95% tolerance ellipse (Q IV-2).

Figure 2 shows the behavior of four female patients with cancer, confirmed by anatomical pathology laboratory, in the tolerance ellipses of the 50th, 75th, and 95th percentiles corresponding to a healthy female population at the characteristic frequency obtained by BIS.

Figure 2 shows the maximum values of Cole's arcs in patients with cervical cancer (stage Ib), breast cancer (stage IIIb), and endometrial cancer (stage IIb). They are within the fourth quadrant of the 50th, 75th, and 95th percentile tolerance ellipses. In contrast, the maximum value of Cole's arc in the patient with cervical cancer (stage IIIb) is in the fourth quadrant of the tolerance ellipses but outside the 95th percentile tolerance ellipse.

Table 3 shows the anthropometric and bioelectric parameters, and the location in the fourth quadrant of the tolerance ellipses, of four female patients, with cancer of different types and stages. This table shows that the patient with cervical cancer (stage IIIb) has higher values of characteristic frequency and electrical resistance at low and high frequencies than the rest. However, the patient with breast cancer (stage IIIb) shows the lowest values of characteristic frequency and characteristic phase angle.

**Table 3.** Anthropometric and bioelectrical parameters of four female patients, with cancer of different locations and stages, diagnosed by anatomical pathology laboratory and obtained by BIS.

Parameters	Patient 1	Patient 2	Patient 3	Patient 4
Pathology	Cervical cancer	Cervical cancer	Endometrial cancer	Breast cancer
Stage	Ib	IIIb	IIb	IIIb
Age (years)	33	55	65	86
Location	Q IV-1	Q IV-3	Q IV-2	Q IV-1
Weight (Kg)	65,50	39,00	64,50	39,00
Height (m)	1,62	1,51	1,47	1,54
R_0_ (Ω)	668,10	871,43	741,24	741,60
R_∞_ (Ω)	478,82	693,48	520,47	498,52
Fc (kHz)	44,48	53,51	49,13	34,64
Φc	9,37	6,49	7,34	5,16

1 Q: quadrant; R_0_: electrical resistance at low frequency; R_∞_: electrical resistance at high frequency; Fc: characteristic frequency; Φc: characteristic phase angle.

**Fig. 3:**
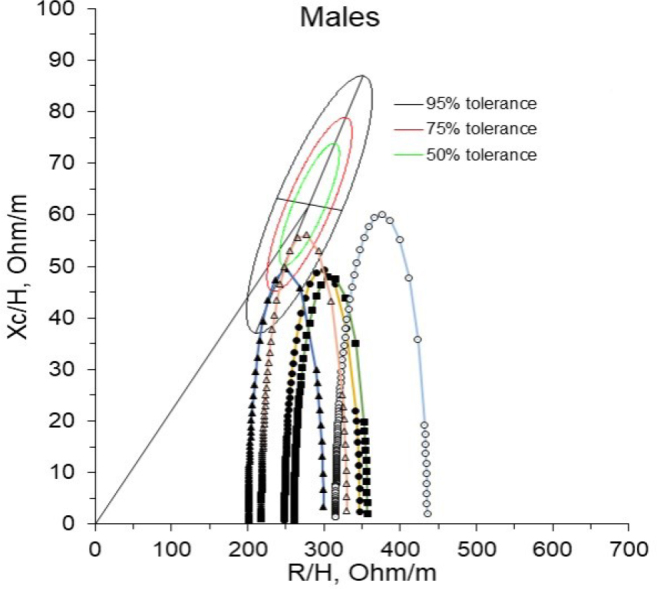
Ellipses of tolerances of a healthy male population at the characteristic frequency; (Δ) reference Cole's arc of a healthy male population, belonging to the fourth quadrant with a maximum value located within the 50% ellipse (Q IV-1). Cole's arcs in four male patients diagnosed with cancer, by anatomical pathology laboratory: (•) Cole's arc of a patient with lung cancer (stage Ib), located in the fourth quadrant and outside the 95% tolerance ellipse (Q IV-3); (▲) Cole's arc of a 58-year-old patient with lung cancer (stage IIIb) located in the fourth quadrant and within the 75% tolerance ellipse (Q IV-1) with a characteristic frequency of 30 kHz; (■) Cole's arc from a patient with melanoma of the skin (stage III) located in the fourth quadrant and outside the 95% tolerance ellipse (Q IV-3); (○) Cole's arch of a patient with colon carcinoma (stage IV) located in the fourth quadrant and outside the 95% tolerance ellipse (Q IV-3).

Figure 3 shows the location of four male patients with cancer, confirmed by anatomical pathology laboratory, in the tolerance ellipses of the 50th, 75th, and 95th percentile, corresponding to a healthy male population at the characteristic frequency.

Figure 3 shows the maximum values of Cole's arcs in patients with lung cancer (stage Ib), skin melanoma (stage III), and colon cancer (stage IV); they are located in the fourth quadrant of the tolerance ellipses but outside the 95th percentile tolerance ellipse. This behavior is more marked in patients with colon cancer.

In contrast, the maximum value of Cole's arc in the patient with lung cancer (stage IIIb) is in the fourth quadrant, within the 75% tolerance ellipse.

Table 4 shows the anthropometric and bioelectrical parameters and the location in the fourth quadrant of the tolerance ellipses of four male patients with cancer of different types and stages.

**Table 4.** Anthropometrics and bioelectrical parameters, obtained by BIS, of four male patients, with cancer of different locations and stages, diagnosed by anatomical pathology laboratory.

Parameters	Patient 1	Patient 2	Patient 3	Patient 4
Pathology	Lung cancer	Lung cancer	skin melanoma	Colon cancer
Stage	Ib	IIIb	II	IV.
Age (years)	58	51	75	30
Location	Q IV-3	Q IV-1	Q IV-3	Q IV-3
Weight (Kg)	75,00	62,00	68,20	52,00
Height (m)	1,75	1,72	1,79	1,64
R_0_ (Ω)	524,59	597,10	638,97	714,04
_∞_ (Ω)	351,23	427,28	468,55	517,07
Fc (kHz)	29,16	42,66	46,15	60,54
Φc	11,21	9,30	8,92	9,08

1 Q: quadrant; R_0_: electrical resistance at low frequency; R_∞_: electrical resistance at high frequency; Fc: characteristic frequency; Φc: characteristic phase angle.

Table 4 shows that the patient with colon cancer (stage IV) has higher values of characteristic frequency and electrical resistance at low and high frequencies than the rest. However, the patient with skin melanoma (stage III) shows a lower characteristic phase angle value than the rest. This last patient has higher values of characteristic frequency and electrical resistance at low and high frequencies than patients with lung cancer.

## Discussion

In Figure 1a, most cancer patients are in the fourth quadrant, within the tolerance ellipses of 50th, 75th, and 95th percentile. They could be associated with less variety in cancer types in this study group. In this sense, in female patients, breast cancer predominates. It could be because breast cancer is the most common type of cancer and the second leading cause of cancer death among women [22]. On the other hand, in Figure 1b a different situation occurs since a greater dispersion of male patients is observed. This behavior could be related to the existence of a greater variety of cancers with different stages of the disease in the analyzed patients of this sex.

In Figure 1, the patients of both sexes who appear within the tolerance ellipses, corresponding to the 50th, 75th, and 95th percentile, could be compensated or in the initial stages of the disease since healthy individuals are normally located in these regions.

The values of R_0_, R_∞_, and Zc in both sexes were higher in patients with suspected cancer compared to healthy individuals. It could be because the extracellular resistance of the tissue is dominant at low frequencies, and the impedance has a large magnitude about the real axis and a small phase angle. As the frequency increases, the capacitance of the cell membrane decreases its reactance, thus decreasing the total magnitude and increasing the phase. Below the characteristic frequency, the magnitude and the phase angle decrease until the reactance becomes negligible at very high frequencies [27].

The Φc is lower in cancer patients since this bioelectrical parameter is positively related to body composition and the clinical and nutritional status in individuals with various diseases such as cancer. This behavior suggests a direct relationship between Φc and this disease since the tumor's extension and evolution determine the patient's metabolic and nutritional impact [28–31].

The analysis of the patients with a diagnosis of the disease confirmed by anatomical pathology laboratory, in the case of the female sex, shows that the maximum value of Cole's arc of the patient with cervical cancer (stage IIIb) is located outside the ellipse tolerance of the 95th percentile. This behavior may be because this type of cancer is advanced at this stage, according to the new classification established by the International Federation of Gynecology and Obstetrics (FIGO) in 2019 [32,33].

This means that the patient's body has suffered severe damage caused by the evolution of the disease itself. These damages include involvement of the lower third of the vagina or pelvic wall, hydronephrosis or nonfunctional kidney, and pelvic or lumboaortic lymph node invasion [34]. Precisely, in advanced stages, the constitutional syndrome (asthenia, anorexia, and weight loss) can appear, as well as severe anemia [35]. All these aspects negatively affect the general health status of this patient and could explain her location outside the 95th percentile tolerance ellipse.

Also, in patients with advanced stages of the disease, the negative impact on patient survival and disease progression of a greater volume, greater extension, and greater tumor dissemination has been demonstrated [36,37].

When observing the behavior of male patients with a diagnosis of the disease confirmed by an anatomical pathology laboratory, it can be seen that there are three patients whose Cole's arcs are located in the fourth quadrant of the tolerance ellipses but outside the 95th percentile tolerance ellipse of the patient. In this case, the colon cancer patient is furthest from the 95th percentile tolerance ellipse. This behavior could be because, in stage IV, colon cancer has spread to other parts of the body, including the liver or lungs; it may be in the lymph nodes [38–41]. At this stage, the patient is in the terminal stage, so he has little opportunity of survival, and his treatment focuses on palliation for most cases [41,42].

In general, cancer patients suffer severe metabolic and pathophysiological changes that contribute to malnutrition. These changes lead to loss of cell integrity, which induces intracellular dehydration and increased extracellular fluid [43,44]. Cellular integrity is essential for the proper functioning of cells, and its alteration has negative consequences on the patient's quality of life. Low Φc values are particularly related to the drastic variations suffered by cell mass, hydration and ionic balance, total body proteins, muscle mass, and general condition in these patients [45–47]. This situation becomes more marked in patients with advanced stages of the disease [48,49].

The extracellular environment affects the low-frequency region, and the intracellular space influences the high-frequency region. Cell membranes have a high capacitance; low frequency currents cannot penetrate the cell and must pass through the extracellular zone. Therefore, these currents are related to low-frequency electrical resistance and extracellular water. This indicates that current flows only through extracellular resistance [50–52].

In contrast, high-frequency currents can penetrate cell membranes and other barriers in the cell structure. In this case, they are related to high-frequency electrical resistance and total body water [50–52].

## Conclusion

The integration of two methods of electrical bioimpedance analysis, BIVA and BIS can be a sensitive complementary tool capable of establishing differences between healthy individuals and cancer patients. This methodology could emerge as a robust indicator of the individual's general health status. It could be enriched by including the analysis of different physiological parameters through estimation equations validated by BIS parameters.

## References

[1] Campa F, Gobbo LA, Stagi S, Cyrino LT, Toselli S, Marini E, Coratella G. (2022). Bioelectrical impedance analysis versus reference methods in the assessment of body composition in athletes. European Journal of Applied Physiology.

[2] Orsso CE, González MC, Maisch MJ, Haqq AM, Prado CM. (2022). Using bioelectrical impedance analysis in children and adolescents: Pressing issues. European Journal of Clinical Nutrition.

[3] Moonen HPFX, Van Zanten ARH (2021). Bioelectric impedance analysis for body composition measurement and other potential clinical applications in critical illness. Current Opinion in Critical Care.

[4] Ceniccola GD, Castro MG, Piovacari SMF, Horie LM, Corrêa FG, Barrere APN, Toledo DO. (2019). Current technologies in body composition assessment: advantages and disadvantages. Nutrition.

[5] Ward LC. (2019). Bioelectrical impedance analysis for body composition assessment: reflections on accuracy, clinical utility, and standardisation. European Journal of Clinical Nutrition.

[6] Silva AM, Campa F, Stagi S, Gobbo LA, Buffa R, Toselli S (2023). The bioelectrical impedance analysis (BIA) international database: aims, scope, and call for data. European Journal of Clinical Nutrition.

[7] Szeszulski J, Lorenzo E, Arriola A, Lee RE. (2022). Community-Based Measurement of Body Composition in Hispanic Women: Concurrent Validity of Dual-and Single-Frequency Bioelectrical Impedance. Journal of Strength and Conditioning Research.

[8] Blue MN, Tinsley GM, Hirsch KR, Ryan ED, Ng BK, Smith-Ryan AE. (2023). Validity of total body water measured by multi-frequency bioelectrical impedance devices in a multi-ethnic sample. Clinical Nutrition ESPEN.

[9] Karava V, Stabouli S, Dotis J, Liakopoulos V, Papachristou F, Printza N. (2021). Tracking hydration status changes by bioimpedance spectroscopy in children on peritoneal dialysis. Peritoneal Dialysis International.

[10] Thanapholsart J, Khan E, Lee GA. (2023). A Current Review of the Uses of Bioelectrical Impedance Analysis and Bioelectrical Impedance Vector Analysis in Acute and Chronic Heart Failure Patients: An Under-valued Resource?. Biological Research for Nursing.

[11] AlDisi R, Bader Q, Bermak A. (2022). Hydration Assessment Using the Bio-Impedance Analysis Method. Sensors.

[12] Matthews EL, Hosick PA. (2019). Bioelectrical impedance analysis does not detect an increase in total body water following isotonic fluid consumption. Applied Physiology, Nutrition, and Metabolism.

[13] Sullivan PA, Still CD, Jamieson ST, Dixon CB, Irving BA, Andreacci JL. (2019). Evaluation of multi‐frequency bioelectrical impedance analysis for the assessment of body composition in individuals with obesity. Obesity Science … Practice.

[14] Park I, Lee JH, Jang DH, Kim J, Hwang BR, Kim S, Lee JE, Jo YH. (2020). Assessment of body water distribution in patients with sepsis during fluid resuscitation using multi-frequency direct segmental bioelectrical impedance analysis. Clinical Nutrition.

[15] Segar JL, Balapattabi K, Reho JJ, Grobe CC, Burnett CM, Grobe JL. (2021). Quantification of body fluid compartmentalization by combined time-domain nuclear magnetic resonance and bioimpedance spectroscopy. American Journal of Physiology-Regulatory, Integrative and Comparative Physiology.

[16] Accardi AJ, Matsubara BS, Gaw RL, Daleiden-Burns A, Heywood JT. (2021). Clinical Utility of Fluid Volume Assessment in Heart Failure Patients Using Bioimpedance Spectroscopy. Front Cardiovasc Med..

[17] Sandini M, Paiella S, Cereda M, Angrisani M, Capretti G, Casciani F, Famularo S, Giani A, Roccamatisi L, Viviani E, Caccialanza R, Montorsi M, Zerbi A, Bassi C, Gianotti L. (2019). Perioperative interstitial fluid expansion predicts major morbidity following pancreatic surgery: appraisal by bioimpedance vector analysis. Annals of Surgery.

[18] Wells JC, Williams JE, Ward LC, Fewtrell MS. (2021). Utility of specific bioelectrical impedance vector analysis for the assessment of body composition in children. Clinical Nutrition.

[19] Stagi S, Silva AM, Jesus F, Campa F, Cabras S, Earthman CP, Marini E. (2022). Usability of classic and specific bioelectrical impedance vector analysis in measuring body composition of children. Clinical Nutrition.

[20] Davydov DM, Boev A, Gorbunov S. (2021). Making the choice between bioelectrical impedance measures for body hydration status assessment. Scientific Reports.

[21] Nwosu AC, Mayland CR, Mason S, Cox TF, Varro A, Stanley S, Ellershaw J. (2019). Bioelectrical impedance vector analysis (BIVA) as a method to compare body composition differences according to cancer stage and type. Clin Nutr ESPEN..

[22] Hong R, Xu B. (2022). Breast cancer: an up‐to‐date review and future perspectives. Cancer Communications.

[23] Jaimes-Morales SA, Aguirre-Cardona VE, Gonzalez-Correa CA. (2024). Ex vivo electrical bioimpedance measurements and Cole modelling on the porcine colon and rectum. Scientific Reports,.

[24] Fu B, Freeborn TJ. (2020). Cole-impedance parameters representing biceps tissue bioimpedance in healthy adults and their alterations following eccentric exercise. Journal of Advanced Research,.

[25] Piccoli A, Pastori G. (2002). BIVA software 2002.

[26] Román AC, Lara A, Morales R, Marañón M, Castillo J, Pérez L. (2021). Base de datos de la caracterización de los parámetros bioeléctricos por métodos de bioimpedancia eléctrica.

[27] Hankinson SJ, Williams CH, Ton VK, Gottlieb SS, Hong CC. (2020). Should we overcome the resistance to bioelectrical impedance in heart failure?. Expert Review of Medical Devices.

[28] Morlino D, Cioffi I, Marra M, Di Vincenzo O, Scalfi L, Pasanisi F. (2022). Bioelectrical Phase Angle in Patients with Breast Cancer: A Systematic Review. Cancers (Basel).

[29] Zhang X, Zhang J, Du Y, Wu X, Chang Y, Li W, Liu Y, Hu W, Zhao J. (2022). The clinical application value of phase angle of six parts in nutritional evaluation of tumor patients. Support Care Cancer.

[30] Amano K, Bruera E, Hui D. (2023). Diagnostic and prognostic utility of phase angle in patients with cancer. Rev Endocr Metab Disord.

[31] Shi J, Xie H, Ruan G, Ge Y, Lin S, Zhang H, Zheng X, Liu C, Song M, Liu T, Zhang X, Yang M, Liu X, Zhang Q, Deng L, Wang X, Shi H. (2022). Sex differences in the association of phase angle and lung cancer mortality. Front Nutr.

[32] Mohamud A, Høgdall C, Schnack T. (2022). Prognostic value of the 2018 FIGO staging system for cervical cancer. Gynecol Oncol.

[33] Van Kol KGG, Ebisch RMF, van der Aa M, Wenzel HB, Piek JMJ, Bekkers RLM. (2023). The prognostic value of the presence of pelvic and/or para-aortic lymph node metastases in cervical cancer patients; the influence of the new FIGO classification (stage IIIC). Gynecol Oncol.

[34] Lecointre L, Lodi M, Molière S, Gantzer J, Eberst L, Menoux I, Le Van Quyen P, Averous G, Akladios C, Baldauf JJ. (2023). Tratamiento del cáncer de cuello uterino en estadio III y IV. EMC-Ginecología-Obstetricia.

[35] D’Oria O, Corrado G, Laganà AS, Chiantera V, Vizza E, Giannini A. (2022). New Advances in Cervical Cancer: From Bench to Bedside. International Journal of Environmental Research and Public Health.

[36] Cho WK, Park W, Kim H, Kim YJ, Kim YS. (2022). Is the pathologic tumor size associated with survival in early cervical cancer treated with radical hysterectomy and adjuvant radiotherapy?. Taiwan J Obstet Gynecol.

[37] Tokalioglu AA, Kilic C, Oktar O, Kilic F, Cakir C, Yuksel D, Comert GK, Korkmaz V, Turan T. (2023). Oncologic outcome in patients with 2018 FIGO stage IB cervical cancer: Is tumor size important?. J Obstet Gynaecol Res.

[38] Liu C, Tian M, Pei H, Tan F, Li Y. (2022). Prognostic Value of the N1c in Stage III and IV Colorectal Cancer: A Propensity Score Matching Study Based on the Surveillance, Epidemiology, and End Results (SEER) Database. J Invest Surg.

[39] Chen H, Yin S, Xiong Z, Li X, Zhang F, Chen X, Guo J, Xie M, Mao C, Jin L, Lian L. (2022). Clinicopathologic characteristics and prognosis of synchronous colorectal cancer: a retrospective study. BMC Gastroenterol.

[40] Ciardiello F, Ciardiello D, Martini G, Napolitano S, Tabernero J, Cervantes A. (2022). Clinical management of metastatic colorectal cancer in the era of precision medicine. CA Cancer J Clin.

[41] Van der Meer R, Bakkers C, van Erning FN, Simkens LHJ, de Hingh IHJT, Roumen RMH. (2023). A propensity score-matched analysis of oncological outcome after systemic therapy for stage IV colorectal cancer: Impact of synchronous ovarian metastases. Int J Cancer.

[42] Wang J, Li S, Liu Y, Zhang C, Li H, Lai B. (2020). Metastatic patterns and survival outcomes in patients with stage IV colon cancer: A population-based analysis. Cancer Med.

[43] Law ML. (2022). Cancer cachexia: Pathophysiology and association with cancer-related pain. Front Pain Res (Lausanne).

[44] Shah UA, Ballinger TJ, Bhandari R, Dieli-Cornwright CM, Guertin KA, Hibler EA, Kalam F, Lohmann AE, Ippolito JE. (2023). Imaging modalities for measuring body composition in patients with cancer: opportunities and challenges. J Natl Cancer Inst Monogr.

[45] Pena NF, Mauricio SF, Rodrigues AMS, Carmo AS, Coury NC, Correia MITD, Generoso SV. (2019). Association between standardized phase angle, nutrition status, and clinical outcomes in surgical cancer patients. Nutr Clin Pract.

[46] Jiang N, Zhang J, Cheng S, Liang B. (2022). The role of standardized phase angle in the assessment of nutritional status and clinical outcomes in cancer patients: a systematic review of the literature. Nutrients.

[47] Ward LC, Brantlov S. (2023). Bioimpedance basics and phase angle fundamentals. Reviews in Endocrine and Metabolic Disorders.

[48] Pereira MME, Queiroz MDSC, de Albuquerque NMC, Rodrigues J, Wiegert EVM, Calixto-Lima L, de Oliveira LC. (2018). The prognostic role of phase angle in advanced cancer patients: a systematic review. Nutr Clin Pract.

[49] Hui D, Moore J, Park M, Liu D, Bruera E. (2019). Phase Angle and the Diagnosis of Impending Death in Patients with Advanced Cancer: Preliminary Findings. The Oncologist.

[50] Moqadam SM, Grewal PK, Haeri Z, Ingledew PA, Kohli K, Golnaraghi F. (2018). Cancer detection based on electrical impedance spectroscopy: a clinical study. J Electr Bioimpedance.

[51] Abasi S, Aggas JR, Garayar-Leyva GG, Walther BK, Guiseppi-Elie A. (2022). Bioelectrical Impedance Spectroscopy for Monitoring Mammalian Cells and Tissues under Different Frequency Domains: A Review. ACS Measurement Science Au.

[52] Catapano A, Trinchese G, Cimmino F, Petrella L, D’Angelo M, Di Maio G, Crispino M, Cavaliere G, Monda M, Mollica MP. (2023). Impedance Analysis to Evaluate Nutritional Status in Physiological and Pathological Conditions. Nutrients.

